# Cervical cancer microbiome analysis: comparing HPV 16 and 18 with other HPV types

**DOI:** 10.1038/s41598-024-73317-8

**Published:** 2024-09-24

**Authors:** Maire Hidjo, Dhananjay Mukhedkar, Collen Masimirembwa, Jiayao Lei, Laila Sara Arroyo Mühr

**Affiliations:** 1 Department of Genomic Medicine, African Institute of Biomedical Science and Technology, 911 Boronia Township, Beatrice, Harare, Zimbabwe; 2 University of Witwatersrand Sydney Brenner Institute for Molecular Biosciences, Johannesburg, 2193 South Africa; 3 Center for Cervical Cancer Elimination, Department of Clinical Science, Intervention and Technology (CLINTEC), Karolinska Institutet, 141 86 Stockholm, Sweden; 4 Hopsworks AB, Åsögatan 119, Plan 2, 116 24 Stockholm, Sweden; 5 Department of Medical Epidemiology and Biostatistics, Karolinska Institutet, 171 77 Solna, Sweden

**Keywords:** Cervical cancer, Human papillomavirus, Cervical microbiome, Metatranscriptome, Tumour virus infections, Microbiome

## Abstract

Differences in the cervicovaginal microbiome may influence the persistence of HPV and therefore, the progression to cervical cancer. We aimed to analyze and compare the metatranscriptome of cervical cancers positive for HPV 16 and 18 with those positive for other HPV types to understand the microbiome’s influence on oncogenicity. RNA sequencing data from a total of 222 invasive cervical cancer cases (HPV16/18 positive (n=42) and HPV “Other types” (n=180)) were subjected to taxonomy classification (Kraken 2) including bacteria, virus and fungi to the level of species. With a median depth of 288,080.5 reads per sample, up to 107 species (38 bacterial, 16 viral and 53 fungal) were identified. Diversity analyses revealed no significant differences in viral or fungal species between HPV16/18 and other HPV types. Bacterial alpha diversity was significantly higher in the "Other HPV types" group for the Observed index (p=0.0074) (but not for Shannon). Cumulative species curves revealed greater species diversity in the “Other HPV types” group compared to “HPV16/18 but no significant differences in species abundance were found between HPV groups. The study did not detect strong significant microbiome differences between HPV 16/18 and other HPV types in cervical cancers. Further research is necessary to explore potential factors influencing the oncogenicity of different HPV types and their interaction with the cervical microbiome.

## Introduction

Human papillomaviruses (HPVs) are a diverse group of double-stranded DNA viruses comprising up to 225 different types, with new types continuously being identified^1–4^. Among these, approximately 12 HPV types are classified as oncogenic, high-risk HPV genotypes, with persistent infection by these types being a necessary cause for cervical cancer.

The oncogenic potential of different high-risk HPV types varies, existing profound differences in carcinogenicity among the HPV types. HPV 16 has by far the highest oncogenic potential, causing more than half of cervical cancers (62.4%), followed by HPV 18 (15.3%)^5^. Besides HPV 16 and 18, there are an additional 5 types (HPV 31, 33, 35, 45, 52 and 58) that are found in >2% of cervical cancers and jointly account for an additional 20% of cervical cancers^5–8^. The least carcinogenic types (HPV 39, 51, 56 and 59) each contribute less than 1% of cervical cancer cases^5^.

These strong differences in carcinogenicity are the reason why some HPV tests analyze for HPV 16 and HPV 18 separately (“high-risk” types) and report an aggregated result “Other HPV” for some other HPV types (oncogenic, probably oncogenic and possibly oncogenic)^5^. Mechanisms on why some HPV types are more oncogenic than others remain not fully understood. It is well known that persistence of the virus is crucial for carcinogenesis, and several authors have reported that persistence can be favored by chronic inflammation in the tissue caused by an imbalanced cervicovaginal microbiome^9,10^. A loss of Lactobacillus genera can lead to the colonization of anaerobic opportunistic bacteria inducing pro-inflammatory cytokine and ROS production, as an example^11,12^. All this evidence positioned microbiome profiles as good candidates to understand the underlying pathogenesis of different types of tumors.

Understanding the interactions between high-risk HPV types and the cervical microbiome is crucial in the era of personalized medicine and targeted cancer prevention strategies. The microbiome may play a significant role in modulating the immune response, influencing viral persistence, and affecting the progression of HPV-induced lesions to malignancy.

In this study, we aimed to analyze and compare the metatranscriptome of cervical cancers caused by HPV 16 and 18 with those caused by other HPV types, aiming to see if the microbiome is distributed differently by HPV type groups.

## Results

All RNA sequencing files corresponding to invasive cervical cancers that were positive for HPV types (n=222) were subjected to taxonomic classification of bacteria, virus and fungi species, and a comparison of species was performed between “HPV16/18” (n=42) and “Other HPV types” (n=180) positive cervical cancers.

### Sequencing coverage and taxonomic resolution

After removing human reads, a median depth of 288,080.5 reads per sample was remaining (range 26,336 - 3,648,489). Taxonomic resolution reached up to 88.41% for bacteria, 88.29% for viruses and 74.07% for fungal species. Filtering by a minimum of 1% of relative abundance, translated into a median of 229,726,5 reads/sample for bacterial species (range 14,530–2,922,113), a median of 4516 reads/sample for viral species (range 638–30,372), and a median of 10,879 reads/sample for fungal species (range 707–63,261).

Up to 107 species (38 bacterial, 16 viral and 53 fungal) showed at least 1% relative abundance and a median of at least 10 reads when considering positive samples. All bacteria and fungi species were shared between HPV16/18 and “Other HPV types” (Supplementary Table 1). Three viral species (3/16) were only detected in “Other HPV types” (2/3 of the species belonged to the *Papillomaviridae* family, and 1/3 was characterized as the phage *Pahexavirus PHL067M01*)

**Fig. 1:**
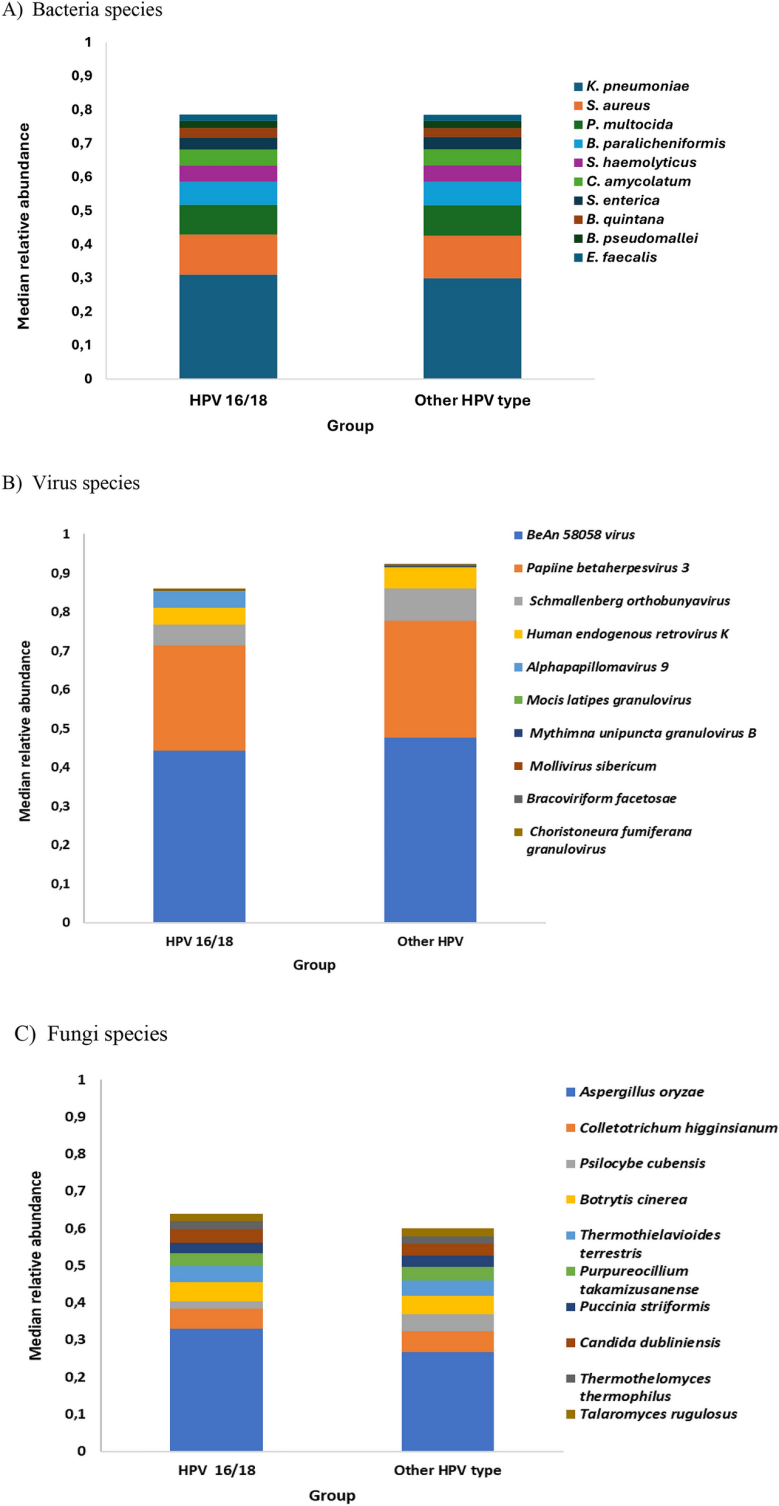
Top 10 microorganism species (median relative abundance) in HPV16/18 and “HPV Other types” cervical cancers.

Most abundant species for bacteria were *Klebsiella pneumoniae, Staphylococcus aureus and Pasteurella multocida* with a median relative abundance of 30.10% among samples, followed by 12.55%, and 9.06%, respectively (Fig. 1). For virus, most abundant species were *Oryzopoxvirus BeAn 58058* virus, *Cytomegalovirus Papiine betaherpesvirus 3*, *and Orthobunyavirus schmallenbergense* with a 47.21%, 28.85%, and 7.36% of median relative abundance, respectively (Fig. 1). Fungal most abundant species were *Aspergillus oryzae, Colletotrichum higginsianum* and *Psilocybe cubensis*, and with a 27.36%, 5.60%, and a 4.16% of median relative abundance, respectively (Fig. 1).

### Diversity analysis

**Fig. 2:**
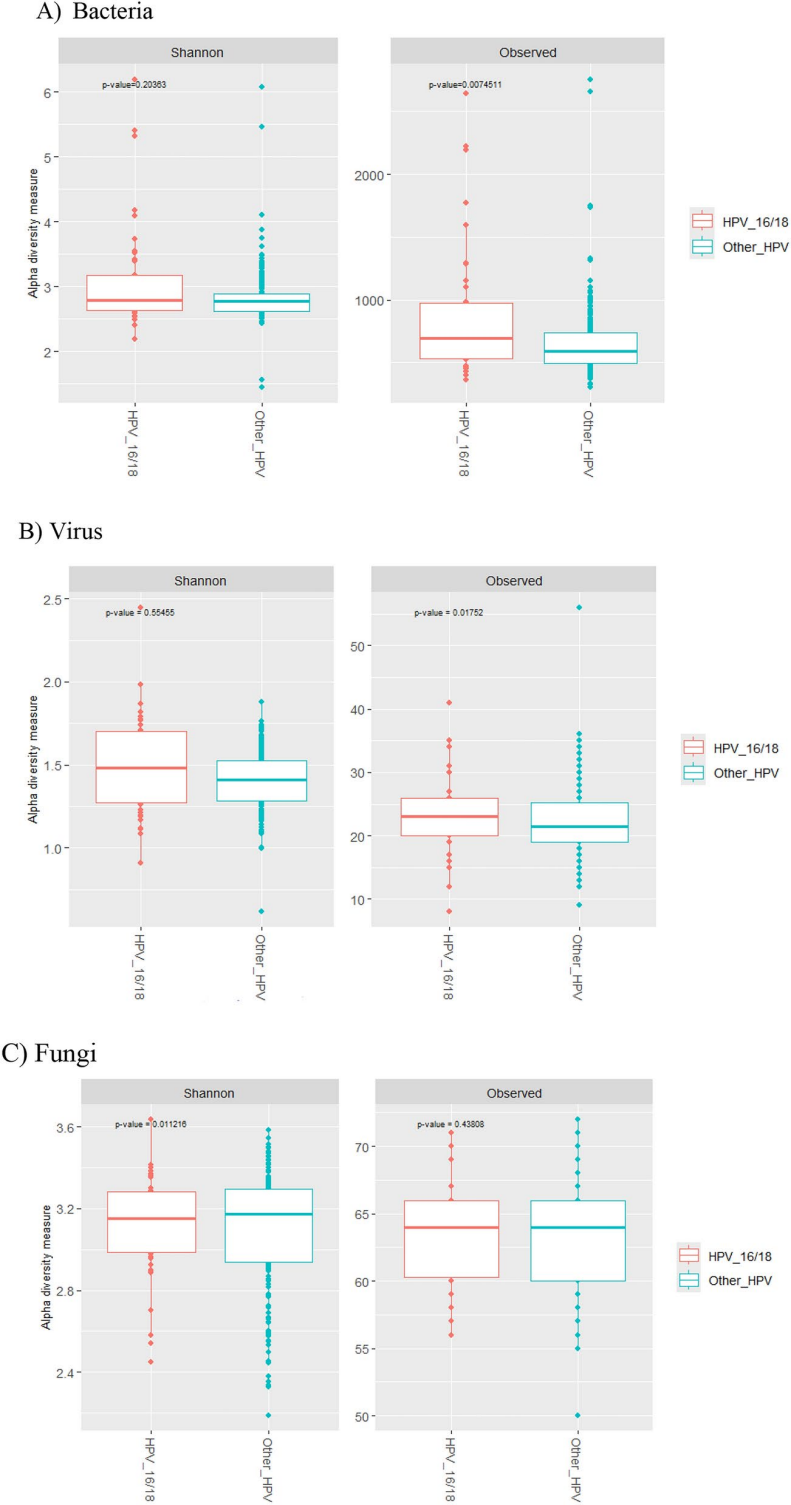
Alpha diversity in “HPV16/18” and “HPV Other types” cervical cancers.

**Fig. 3:**
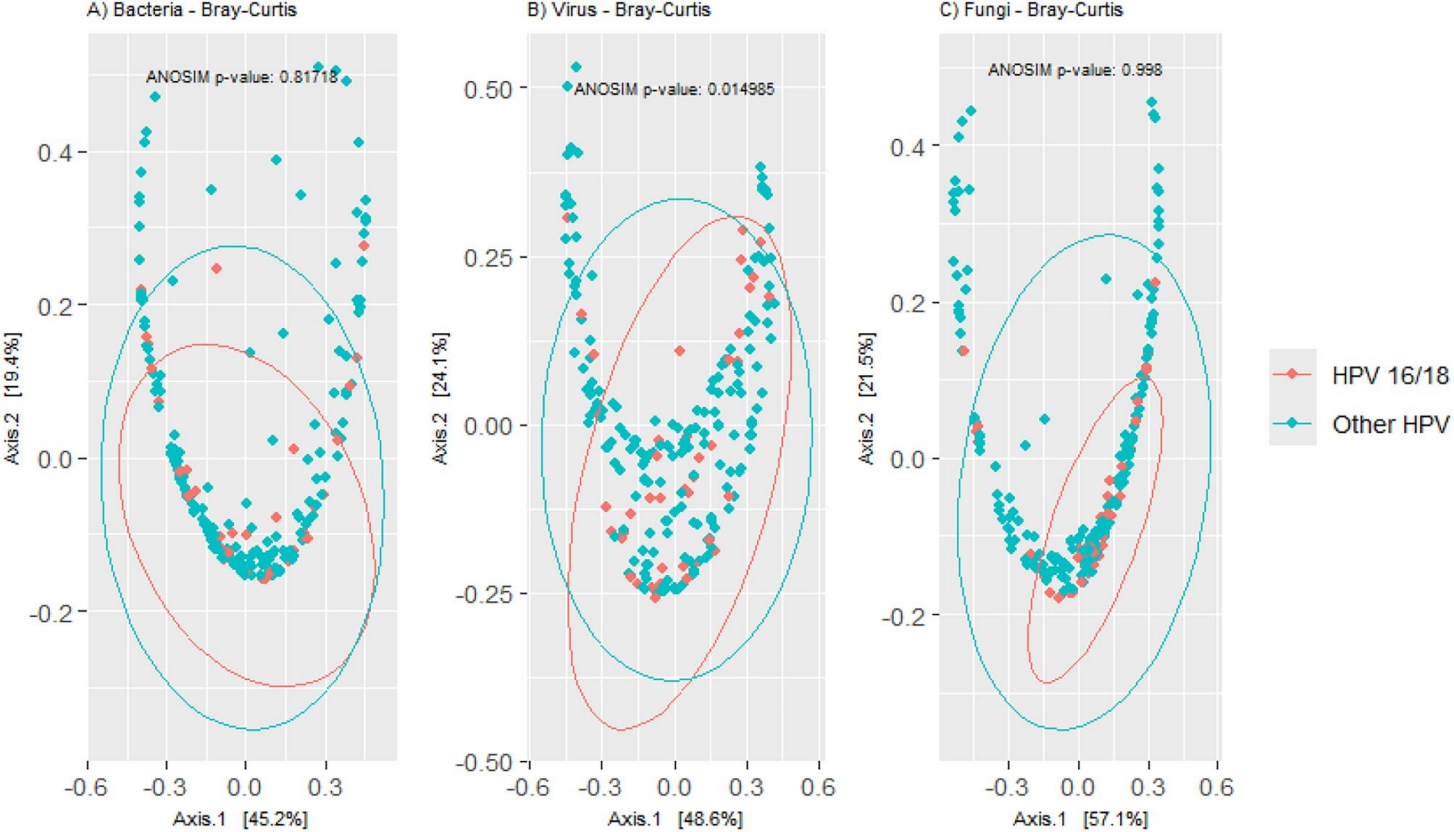
Beta diversity analysis in cervical cancers by HPV type groups.

Alpha and beta diversity analyses revealed no significant differences between the “HPV16/18” and “Other HPV types” groups for viral and fungal species (Figs. 2, 3). For bacteria, however, a significant difference was observed in alpha diversity: the “Other HPV types” group had a higher alpha diversity compared to the “HPV16/18” group, as indicated by the Observed index (p=0.0075).

In contrast, cumulative species curves, which plot the cumulative number of species detected against the number of samples, indicated a higher species count in the “Other HPV types” group across all three domains (bacteria, viruses, fungi). This suggests greater species diversity in the “Other HPV types” compared to “HPV16/18” (Supplementary Figs. 1–3).

Age and FIGO stage showed no significant differences when comparing HPV16/18 and “Other HPV types” positive cervical cancers. (p=0.0278 and p=0.6254, respectively). No differences in number of species nor sequencing depth for bacterial (p=0.1310 and p=0.8259, respectively) and fungal species (p=0.5093 and p=0.8887, respectively) were observed when comparing HPV16/18 and “Other HPV types” positive cervical cancers. For viruses, there were no significant differences when comparing sequencing depth (p=0.3320) but there were significant differences when comparing number of viral species between HPV 16/18 and “Other HPV types” cervical cancer groups (p=0.0050). Therefore, sequencing depth was used to adjust differential abundance models for viral species.

### Differential abundance

Differential abundance analysis was conducted with a cutoff set at 1% relative abundance and a median of at least 10 reads among the samples where the species were present. A total of 107 species (38 bacterial, 16 viral and 53 fungal) were subjected for the abundance analysis. The viral analysis was adjusted by the number of species (due to presence of significant p-values).

Differential abundance analysis showed no particular species being significantly abundant in any of cervical cancer groups (HPV 16/18 vs HPV “Other types”) (Supplementary Table 1).

## Discussion

We report the metatranscriptomes identified when analyzing cervical cancers based on their HPV type positivity (HPV16/18 vs other HPV types), including the identification of bacteria, viruses, fungi at a species level. We detected 107 different species (38 bacterial, 16 viral and 53 fungal).

The study possesses several significant strengths: (1) comprehensive RNA Sequencing analysis we conducted a thorough analysis of RNA sequencing data, aiming to identify all transcriptionally active bacteria, viruses, and fungi down to the species level, and (2) implementation of stringent cut-offs. To fortify the robustness of our analysis, we included different metrics for diversity analysis (Shannon and Observed), analysed different metadata variables to see if these could influence final results, performed normalization, and transformation techniques to effectively address data sparsity using a threshold at 1% relative abundance as well as required a median of at least 10 reads among the samples where the species were present. We aimed to reduce complexity, noise and technical variability while preserving data integrity and representing main communities. Indeed, up to 67 species identified showed at least 1% relative abundance but less than a median of 10 reads among samples where they were present (data not shown). Such a low amount raises uncertainty regarding the validity of their presence.

Our analysis revealed an average of 65.77% zero counts per bacterial taxon and 95.88% zero counts per viral taxon in our dataset. These high percentages of zero counts are indicative of zero-inflation, a common phenomenon in microbiome data where many taxa are absent from a significant proportion of samples. This zero-inflation can impact the accuracy of traditional diversity metrics and rarefaction curves, which may be sensitive to low-abundance data and yield unstable or misleading results. Given these challenges, we employed cumulative species curves as an alternative method for assessing species accumulation and diversity. The cumulative curves suggested higher species counts in the 'Other HPV types’ group compared to the 'HPV16/18’ group, indicating greater species diversity. In contrast, alpha and beta diversity metrics did not reveal statistically significant differences between the groups. To investigate deeper and gain a more comprehensive understanding of the microbial community dynamics, we utilized a tailored analytical approach, metagenomeSeq for differential abundance testing. This method is more robust in handling zero-inflated data and allowed us to detect differences that might have been overlooked by traditional methods. No particular species was found to be significantly abundant in any of cervical cancer groups. These findings underscore the complexity of microbial diversity analysis and highlight the necessity of using multiple metrics to fully capture and understand the microbial diversity present in different HPV groups. In addition, correlation analysis could offer additional insights into the relationships between different taxa, potentially highlighting interactions or dependencies within the microbiome.

While the study boasts several strengths, it’s important to acknowledge one potential limitation: the nature of the specimens. Specimens sequenced were FFPE material, and that carries a higher risk of DNA degradation (translating into lower DNA fragments and biased amplification) and contamination. Nevertheless, these FFPE specimens had previously been sequenced as described, with blank paraffin controls sectioned after each cervical tumor FFPE sample to control for contamination and the presence of environmental communities (a common occurrence when performing multiple comparisons). As an example, most abundant bacterial species detected were *Klebsiella pneumoniae* (30.10% relative abundance), *Staphylococcus aureus* (12.55%) and *Pasteurella multocida* (9.06%), with no differential abundance being significant between both HPV groups (HPV 16/18 vs. other HPV types). The identification of Klebsiella, Staphylococcus, Pasteurella in paraffin blank blocks was only achieved in 1/11^13^, suggesting that these bacteria are likely to be originating from the samples themselves rather than from environmental contamination.

Bacterial (and in a few cases viral) communities have already been analyzed and compared among normal cervical tissue (no lesion) and different lesion grades as well as invasive cervical cancer. Ure et al. investigated metatranscriptome differences between HPV-positive and HPV-negative cervical cancers and reported higher bacterial diversity and lower abundance of Lactobacillus in cervical cancers^13^. Their analysis investigated bacteria and viral communities up to genus level and revealed no significant microbiome differences between HPV-negative and HPV-positive cervical cancers^13^. Using a subset of these specimens, (HPV positive cancers) we aimed to conduct a deeper exploration, going down to the species level and including fungi communities, and see if differences were seen in the microbiome when comparing HPV 16/18 vs “Other HPV types”. We did not find any statistical difference between the metatranscriptomes when comparing HPV groups, suggesting that while the microbiome may play a role in HPV persistence, as some authors report, it does not appear to be influenced by the specific HPV type.

One could further argue that there are also differences in oncogenicity among the "Other HPV” types. There were up to 21 different HPV types detected among the “Other HPV types”, with 12/21 types being found in five or fewer specimens. The low frequency of many of these genotypes reduces the statistical power of any analysis, making it difficult to draw meaningful conclusions from the data, and therefore, further discrimination of types was not performed. It is noteworthy that among the 2850 samples genotyped, only 92 showed multiple infections. For the samples initially reported as “apparently HPV negative” (n=223) and those that were HPV positive upon sequencing (n=169), each sample had reads corresponding to a single HPV type only (single infection).

Many HPV studies are done on comparisons between HIV positive and HIV negative subjects. The HIV status of the cohort in this study is not known. Worldwide, approximately 5% of all cervical cancer cases are attributable to HIV. However, the fraction of cervical cancer cases related to HIV in Sweden is expected to be far below 5%. According to earlier published Swedish data^14–16^, nearly all women (96%) living with HIV were receiving antiretroviral therapy, and of whom 97% had suppressive antiretroviral therapy. Additionally, a significant majority (87%) had a CD4 count above 350, demonstrating an exceptionally well-managed HIV cohort in Sweden.

Understanding the differences in oncogenicity and identifying factors that influence prognosis is crucial, especially considering the impact of vaccination on preventing the most oncogenic types of cancer. Our study may serve as a baseline for species comparison when analyzing cervical cancers (especially in similar settings). We did not detect microbiome differences when analyzing RNA sequencing data from HPV 16/18 versus other HPV types in cervical cancers. Further studies could be valuable in better understanding why different HPV types show differences in oncogenicity.

## Methods

### Sample collection

Samples collected belonged to a systematic, population-based HPV genotyping of invasive cervical cancers, as described in Lagheden et al.^17^.

Briefly, all cases of invasive cervical cancer occurring in Sweden during 2002-2011 were identified (n=4254). A total of 2850 cervical cancer formalin-fixed paraffin-embedded (FFPE) specimens (one block per patient) were collected. Extraction was performed using a xylene-free protocol as previously described^18^, and stored at – 20 °C if not immediately used for experiments. Specimens were HPV typed using a PCR with modified general primers (MGP)-PCR (primer targeting L1) and hybridisation with type-specific probes in Luminex^19,20^. In case of specimens revealing HPV negativity, samples were further subjected to a qPCR targeting E6/E7 genes of HPV 16/18. Cervical cancer specimens that were still HPV negative after both assays (394/2850) (together with a subset of 59 HPV positive samples, used as positive controls) were whole genome sequenced (NovaSeq 6000 system (Illumina, USA)^21^. For this study, we retrieved the fastq files from all specimens that showed HPV sequencing reads (HPV positive samples) when subjected to whole genome sequencing (n=222) and grouped them as HPV16/18 positive (n=42) and HPV “Other types” (n=180). Characteristics of the patients and the primary invasive cervical cancers by HPV type group can be seen in Table 1.

**Table 1:** Clinical information for patients and primary invasive cervical cancers by HPV type group.

Group	HPV positive cervical cancers	HPV16/18	“Other HPV types”
Sample size	222	42	180
Age median (Min-Max)	56.5 (24–95)	50 (25–88)	58 (24–95)
HPV species	Alpha 1, n=3	Alpha 7, n=13	Alpha 1, n=3
	Alpha 5, n=1	Alpha 9, n=29	Alpha 5, n=1
	Alpha 6, n=11		Alpha 6, n=11
	Alpha 7, n= 49		Alpha 7, n= 36
	Alpha 9, n= 128		Alpha 9, n= 99
	Alpha 11, n=27		Alpha 11, n=27
	Alpha 13 n=1		Alpha 13 n=1
	Beta 2, n=1		Beta 2, n=1
	Gamma 11, n=1		Gamma 11, n=1
FIGO Stage	IA, n=47	IA, n=7	IA, n=40
	IB, n=78	IB, n=15	IB, n=63
	II, n=47	II, n=9	II, n=38
	III+, n=50	III+, n=11	III+, n=39
Histologic type	SCC, n=202	SCC, n=29	SCC, n=173
	ASC, n=2	ASC, n=2	ASC, n=0
	AC, n=14	AC, n=8	AC, n=6
	Others, n=4	Others, n=3	Others, n=1

*AC* Adenocarcinoma, *ASC* Adenosquamous cell carcinoma, *FIGO* International Federation of Gynecology and Obstetrics stage, *SCC* Squamous cell carcinoma.

Sequencing of these specimens had already been performed^21,22^ following the SMARTer^®^ Stranded Total RNA-Seq Kit v2 - Pico Input Mammalian library preparation guide (Takara, US), omitting the fragmentation step and using the NovaSeq 6000 system (Illumina, USA) at 2 × 150 bp, in seven different runs (one run per group), aiming for 30 M paired end reads/sample^21,22^. We collected the raw fastq files as well as the metadata for this study (n=222).

### Sequencing data pre-processing

Raw fastq files were first subjected to quality assessment and adapter trimming using Trimmomatic^23^, with a minimum read length set to 18 bp. High-quality reads were mapped against the human reference genome GRCh38 using NextGenMap^24^, retaining only those reads that did not align to the human genome with more than 95% identity over 75% of their length for further microbiome analysis. Subsequently, high-quality non-human reads were taxonomically classified using Kraken2 v. 2.1.1^25^, against a reference database that included all RefSeq bacterial, viral, and fungal genomes as of January 2023, with a confidence threshold of 0.1.

### Downstream diversity analysis and statistics

In R (v.4.2.2), biom files generated from Kraken2 reports were imported along with sample metadata to create a phyloseq object^26^. Further analysis was performed using the tidyr^27^, ggpubr^28^, and vegan^29^ R packages.

Diversity analyses were conducted separately for each taxonomy group with statistical significance defined as a p-value < 0.01. Alpha diversity metrics, such as observed species and the Shannon index, were calculated after rarefaction to 18361 reads for bacteria, 659 reads for viruses, and 742 reads for fungi. Differences in alpha diversity were evaluated using the Mann-Whitney test. For beta diversity analysis, the Bray-Curtis index was used to analyze differences between communities, which were visualized using principal component analysis and assessed with analysis of similarities (ANOSIM) tests.

To further assess species accumulation, cumulative species curves were generated for each taxonomic group (bacteria, viruses, fungi). These curves plot the cumulative number of species detected against the number of samples, offering insights into how species richness accumulates with increasing sample size. This approach complements traditional alpha and beta diversity metrics and addresses some limitations of rarefaction, such as sensitivity to low-abundance data.

Metadata, including age (below vs. above median age), FIGO stage (confined to the cervix [IA and IB] vs. spread beyond the cervix [II and III]), sequencing depth, and number of species were analyzed to determine if there were differences between HPV groups (HPV16/18 vs. “Other HPV types”) using the Mann-Whitney test. Variables described above that showed statistically significant differences were used to adjust differential abundance models.

Differential abundance analysis was carried out using metagenomeSeq^30^, with a cutoff of 1% relative abundance and a median of at least 10 reads among the samples where the species were present. Species counts were transformed using cumulative sum scaling (CSS), log2 transformation, and pseudocount addition. Models for each taxonomic group were adjusted for variables with significant ANOSIM p-values, and the Benjamini-Hochberg method was applied for adjusting p values to control the false discovery rate.

## Supplementary Information

Supplementary Figures.

Supplementary Table 1.

## Data Availability

All sequencing files (non-human sequences) used in the present study are publicly available at the Sequence Read Archive (SRA) within the bio-project ID PRJNA563802.
