# Supplementary material for: Cervical cancer microbiome analysis: comparing HPV 16 and 18 with other HPV types
Source: Sci Rep. 2024 Sep 24;14:22014. doi: 10.1038/s41598-024-73317-8 (PMC11422507; doi:10.1038/s41598-024-73317-8)

Supplementary Figures

Cervical Cancer Microbiome Analysis: Comparing HPV 16 and 18 with Other HPV Types

Maire Hidjo, Dhananjay Mukhedkar, Collen Masimirembwa, Jiayao Lei, Laila Sara Arroyo Mühr

Content

Supplementary Figure 1 Page 2

Supplementary Figure 2 Page 2

Supplementary Figure 3 Page 3

Supplementary Figure 1: Cumulative species curve for bacteria

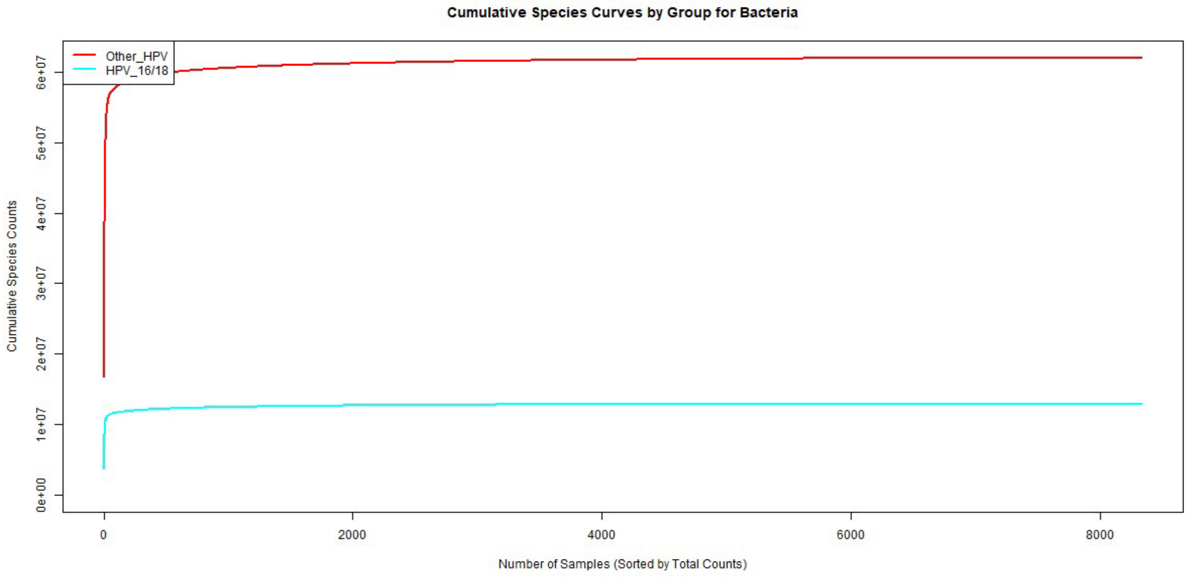

Supplementary Figure 2: Cumulative species curve for virus

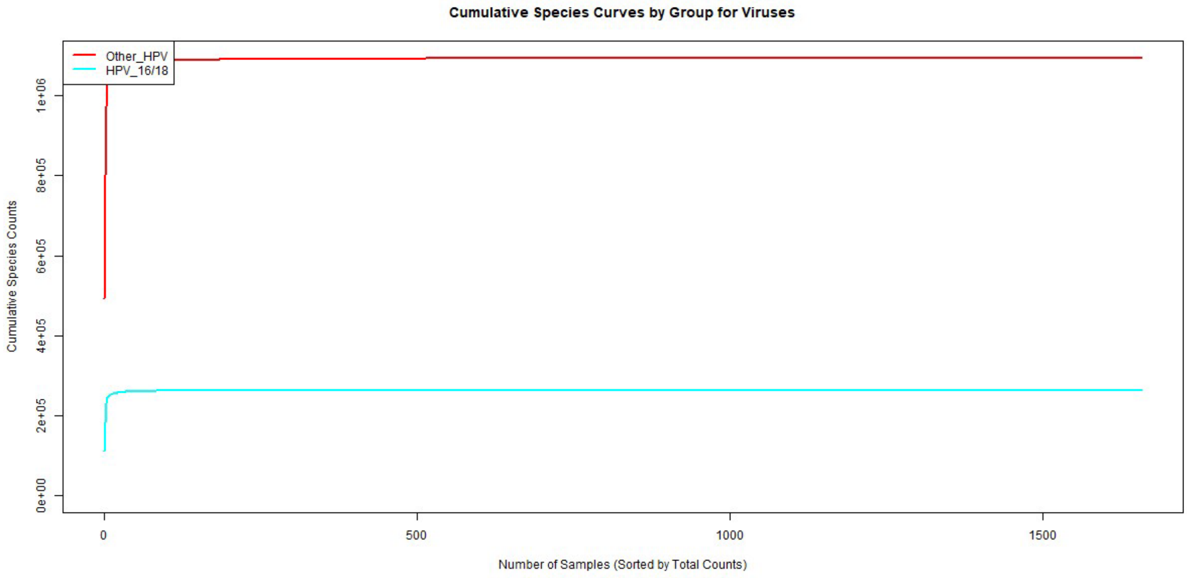

Supplementary Figure 3: Cumulative species curve for fungi

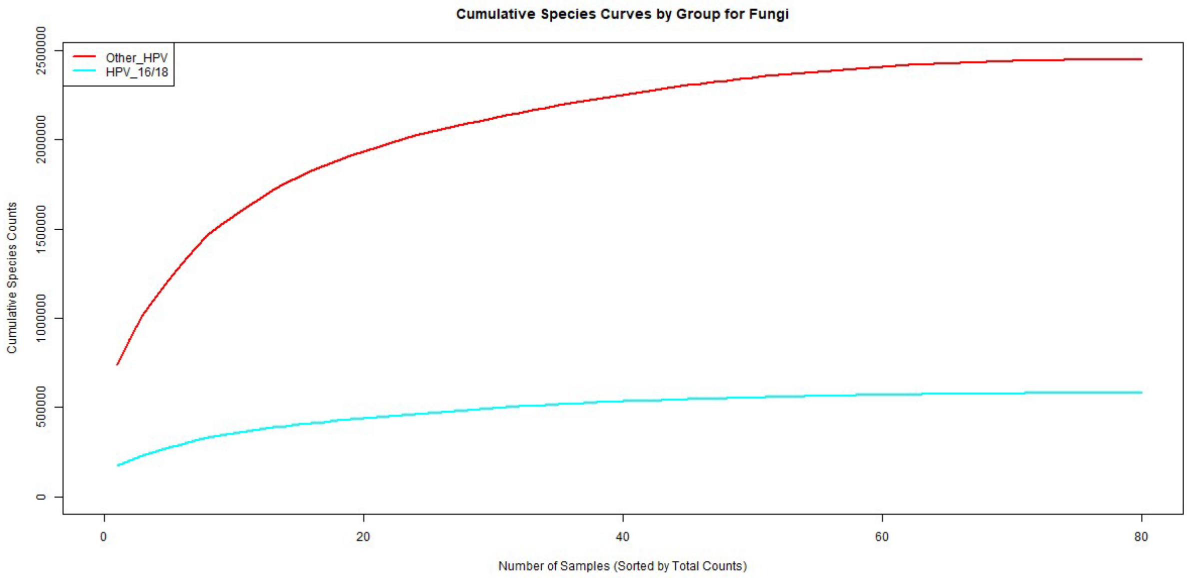

Supplement: Supplementary file 1 — Supplementary Figures. [file 41598_2024_73317_MOESM1_ESM.pdf]
